# Mild hypoxia and human bone marrow mesenchymal stem cells synergistically enhance expansion and homing capacity of human cord blood CD34+ stem cells

**DOI:** 10.22038/IJBMS.2018.26820.6561

**Published:** 2018-07

**Authors:** Fatemeh Mohammadali, Saeid Abroun, Amir Atashi

**Affiliations:** 1 Department of Hematology and Blood Banking, Faculty of Medical Sciences, Tarbiat Modares University, Tehran, Iran; 2 Stem cell and Tissue Engineering Research Center, Shahroud University of Medical Sciences, Shahroud, Iran

**Keywords:** CD34+ cells, Cord blood, CXCR4, Hematopoietic stem cell, Hypoxia, Mesenchymal stem cell

## Abstract

**Objective(s):**

Cord blood (CB) is known as a valuable source of hematopoietic stem cells (HSC). Identifying strategies that enhance expansion and maintain engraftment and homing capacity of HSCs can improve transplant efficiency. In this study, we examined different culture conditions on *ex vivo* expansion and homing capacity of CB-HSCs.

**Materials and Methods:**

In this study, 4-5 different units of human CB in each of 3 independent experiments were collected.CD34+ HSC was isolated, cultured in the serum-free medium(Stem line II) and supplemented with cytokines: FMS-like tyrosine kinase 3 ligand (FLt3L), Thrombopoietin (TPO), stem cell factor (SCF) with/without bone marrow mesenchymal stem cell (BM-MSC) feeder layer in normoxia (20% O_2_) and mild hypoxia (5% O_2_) for 7 days. Before and after this period, total nucleated cell count (TNC), CD34+ cells count, Colony-forming cell (CFC) assay, migration assay and CXCR4 expression were evaluated by real time PCR. Data analysis was performed with t- test and ANOVA. P-value less than 0.05 was considered as statistically significant differences.

**Results:**

At the end of 7 days of culture, the highest count of TNC, CD34+ cells, CFUs, migration percentage and CXCR4 mRNA level were observed in feeder+cytokine group at 5% O_2_ tension. Our findings demonstrated statistically significant (1.7-3.2 fold) increase of *CXCR4* gene expression in hypoxia versus normoxia.

**Conclusion:**

Combination of BM-MSC and mild hypoxia (5% O_2_) not only improves HSC expansion but also enhances homing capacity of HSC and better mimickes the niche microenvironment conditions.

## Introduction

Stem cells are known for their ability to maintain self-renewal and undifferentiated state that preserves them throughout adult life (1-4). Hematopoietic stem cells (HSCs) can be obtained from various sources such as cord blood (CB). Human CB contains about 0.02 -1.43 % CD34+ HSCs with high proliferative potential (5).

Besides the advantages of CB, including widespread availability and lower rate of graft-versus-host disease (GVHD), CB also has some disadvantages, including the low number of cells collected in one unit, which can be less than the amount needed for engraftment in adults, and the slower engraftment time of platelets and neutrophils (6-8).

There are several approaches to overcome the low number of HSC in CB. Expansion methods of hematopoietic stem /progenitor cells and culture with feeder layer were suggested as the most suitable ways, although due to our limited knowledge about factors that maintain stem cell self-renewal, these techniques are challenging. Among the different stimulatory cytokines identified, cocktail of stem cell factor (SCF), FMS-like tyrosine kinase 3 ligand (FLt3L), and Thrombopoietin (TPO) seems to better maintain HSC self-renewal (9).

*In vivo* HSC is reside in a specific microenvironment known as ‘‘niche’’. Multiple cellular types, soluble and membrane bound factors and extracellular matrix components form this niche (10).

Mesenchymal stem cells (MSCs) in stem cell niches support the expansion, quiescence and differentiation of HSCs (11). Several studies have shown that bone marrow derived MSCs (BM-MSC) secrete cytokines including interleukin-6 (IL-6), IL-7, IL-8, IL-11, IL-12, IL-14, IL-15, macrophage-colony stimulating factor (M-CSF), SCF and FLt3L (12).

Several studies demonstrated that stem cell niches are located in the low O2 tension environment, far from blood vessels (13). Studies in murine and human HSCs demonstrated that HSC culture at 20% O_2_ increases the exhaustion of stem cells, while culture in anoxic conditions (0.1-1% O_2_) better maintains stem cell quiescence (14), and culture at higher O_2_ tensions (3-5 %) maintains cell proliferation beside the preservation of self-renewal (15-17). 

It is presumed that mechanisms by which HSC respond to hypoxia is related to the hypoxia inducible factor-1(*HIF-1*) protein (18), which is stabilized under low O_2_ levels (<5%) and degraded under normoxic environment. 

Hematopoietic engraftment after HSC transplantation is dependent on proliferation of primitive repopulating cells and homing capacity (19). Identifying strategies to enhance expansion and maintain homing capacity of HSCs can improve transplant efficiency, particularly when HSC number is limited. The chemokine receptor *CXCR4* and its ligand, stromal cell-derived factor 1 (*SDF-1*), is important for homing of HSCs to the bone marrow (20). Studies have shown that* HIF1α*, which is induced by hypoxic conditions, affects the expression of *SDF-1* (21). In ischemic sites of injury, *HIF1α* induced expression of *SDF-1* and enhanced the migration and homing of circulating *CXCR4*+ progenitor cells (22). 

Considering the mentioned studies, we presumed that combining the BM-MSC and mild hypoxia (5 % O_2_) as a novel culture system would promote *ex vivo* expansion and homing of HSCs.

## Materials and Methods

***CB collection and CD34+ cells purification***

CB samples were collected from umbilical CB of 4-5 different full term deliveries after taking parent’s written informed consent in each experiment (N=3). CB units were obtained from the Iranian Blood Transfusion Organization. Briefly, up on delivery of baby the cord was doubly clamped, transected and the blood was collected in bags containing citrate phosphate dextrose (CPD) anti-coagulant. After collection, the samples were stored at room temperature (18-24 °C) and processed within 12 hr. This research was approved by the Ethics Committee at Tarbiat Modares University with reference number 4506. Red cells were precipitated with hydroxyethyl starch (HES solution 6%, Grifols, Spain). Mononuclear cells (MNCs) fraction was isolated using density gradient centrifugation on Ficoll-Hypaque (Lymphodex, Inno-Train, Germany). CB-CD34+ cells were purified from MNCs by MACS CD34+ cell isolation kit following the manufacturer’s instructions (Miltenyibiotec, USA CD34 MicroBead Kit)*.* CD34+ cell purity was evaluated by flowcytometry analysis using FITC- human CD34 antibody (BD Pharmingen)*.* Non-specific reactions were excluded using isotype controls*.*

***Feeder layer preparation***

**Figure 1:**
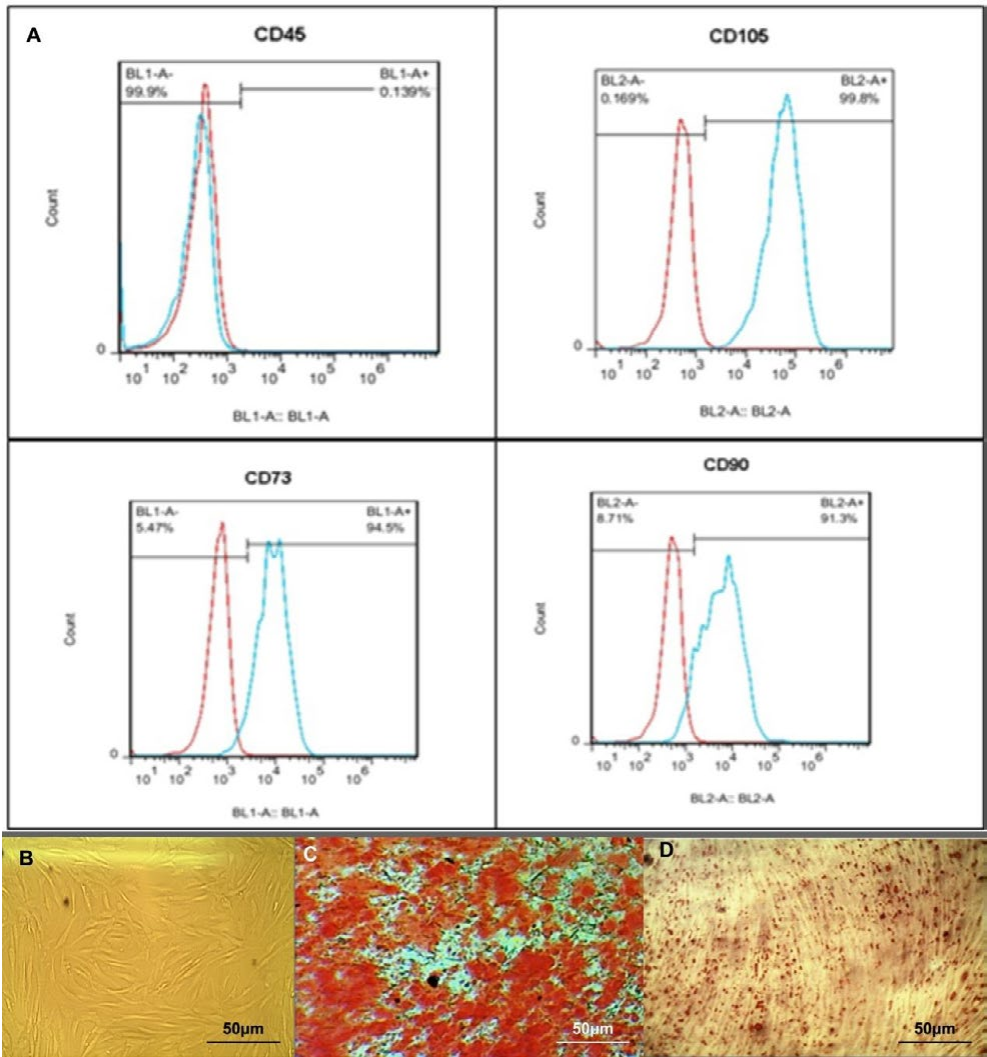
*In vitro* characterization of mesenchymal stem cells from human bone marrow (BM-MSCs). (A) Flowcytometry analysis results showed that BM-MSCs were positive for MSC markers CD90, CD105 and CD73, but were negative for the pan- leukocyte marker CD45. (B) Isolated BM-MSCs showed a spindle-like morphology under bright field microscopy. Osteogenic (C) and adipogenic (D) differentiations of BM-MSC were confirmed by Alizarin Red S staining and by Oil Red o staining, respectively. Scale bar: 50 μm

BM-MSCs were taken from Stem Cells and Tissue Engineering Department, Stem Cell Technology, Tehran, Iran. Three BM-MSC samples from different healthy donors were cultured in Dulbecco’s Modified Eagle’s Medium(DMEM) with 10% fetal bovine serum (FBS, GIBCO, USA) supplemented with Streptomycin (0.025 mg/ml) and Penicillin (0.025 U/ml; GibcoBRL), at 37 ^°^C and 5% CO_2_ in a humidified atmosphere. When cells reached 80% confluency, the cells were trypsinized and seeded at about 1×10^4^ cells/cm^2^. Cells were expanded for 2-4 passages. Phenotypic characterization and differentiation capability to osteoblasts and adipocytes was carried out (Figure 1). MSCs were characterized by monoclonal antibodies against CD90, CD105, CD73 and CD45. MSCs were positive for CD90, CD105, and CD73, and were negative for CD45. Cells were seeded on 24-well plates (1×10^4^ cells/ well) and used as a feeder layer and cultured until 70-80% confluency.

***Coculture with feeder layer***

Isolated CD34+ cells were seeded at a density of 1×10^4^ /well on 24-well plates in stemline II serum free media (Sigma, Germany) supplemented with the combination of human recombinant cytokines: Flt3-L (50 ng/ml, ORF Genetics, Iceland), SCF (50 ng/ml ,ORF Genetics, Iceland) and TPO (50 ng/ml, Peprotech, Rocky Hill, NJ, USA). In this experimental study, the cells harvested at 2–4 passages were used as BM-MSCs. All experiments were cultured at 37^ °^C, 5% CO_2_ in hypoxia (5% O_2_, two gases incubator) and normoxia (20% O_2_), for 7 days. The 6 different groups of culture conditions were as follows:

1-Cytokine group: CD34+ cells cultured in the Stemline II serum-free media in presence of the mentioned cytokines in 20% O_2_.

2-Feeder group: CD34+ cells cultured in the Stemline II directly on the confluent MSC feeder layer without the mentioned cytokines in 20 % O_2_.

3-Feeder +cytokine group: CD34+ cells cultured in the Stemline II on a confluent MSC feeder layer and in the presence of the mentioned cytokines in 20% O_2_.

4-Cytokine group: CD34+ cells cultured in the Stemline II in presence of the mentioned cytokines in 5% O_2_.

5-Feeder group: CD34+ cells cultured in the Stemline II directly on the confluent MSC feeder layer without the mentioned cytokines in 5% O_2_.

6-Feeder +cytokine group: CD34+ cells cultured in the Stemline II on a confluent MSC feeder layer and in the presence of the mentioned cytokines in 5 % O_2_.

Additionally, as a control group, HSCs were cultured in Stemline II without any cytokines and MSC layer. After 7 days, the cultures were harvested and the number of viable cells counted using trypan blue staining and total number of nucleated cells was counted.

***Flow cytometric analysis***

1×10^5^ CD34+ cells from 6 different culture conditions were collected and resuspended in phosphate-buffered saline. After labeling the cell suspension with monoclonal antibodies (CD34-FITC, BD Pharmingen™, USA), the cells were incubated for 30-45 min on ice. Expansion fold of CD34+ cells was calculated by comparing total nucleated cell count (TNC× CD34^+^ percentage) obtained after 7 days with TNC× CD34^+^ percentage before culture initiation. Data analysis was performed using FlowJo software (Tree Star, Inc, Ashland, OR). 

***In vitro migration assay***

Filters 6.5 mm diameter Transwell with 5 µm pore size (Costar, Cambridge, MA, USA) was used for migration assays. A total of 1×10^4^ fresh CD34+ cells and harvested cells from different culture conditions were cultured in 100 μl serum free medium (Stemline II) in upper chamber of a 24 well plate, and 600 μl serum free medium in the presence of 100 ng of *SDF 1α*, (Strathmann Biotech GmbH Hannover, Germany) was added to the lower chamber, then incubated for 4 hr at 37 ^°^C and 5 % CO_2_ to allow migration. After 4 hr, migrated cells were harvested and counted manually using hemocytometer. The percentage of migrating cells was calculated using input cells. As a control group, one set was run without the addition of* SDF1α* to evaluate spontaneous migration.

***Colony-forming cell (CFC) assay***

 1×10 3 of fresh CD34+ cell or harvested cells from 6 different culture conditions at day 7 were cultured on 12-well plates in a cytokine-supplemented methylcellulose media (MethoCultH4434 classic with cytokine, Stem cell Technologies, Canada) + 2 % FBS in Iscove’s Modified Dulbecco’s Medium (IMDM) following the manufacturer’s instructions to enumerate CFCs. After 14 days of incubation at 37 ^°^C under humidified conditions in 5 % CO_2_, CFU colonies consisting of more than 50 cells were counted. The experiments were performed in duplicate in 3 independent experiments. The total CFU fold change was calculated by dividing the number of colonies obtained per 1×10^3^ cultured cells by the number of the colonies obtained at day 0 and multiplying by the fold increase in the total number of cells.

***RNA extraction and CDNA synthesis***

RNA of the cells was extracted by TRIzol (Sigma, Germany), then the total RNAs were reversely transcribed using the cDNA Kit (Primescript RT reagent kit, Perfect Real Time, Takara), following the manufacturer’s instructions. All cDNA samples were run in duplicate for target (*CXCR4*) gene or endogenous control (*HPRT*). 

***Real time PCR***

Real time PCR was performed on 1 μl total cDNA in 10 μl reaction volume with 0.3 μM of each primer and 5 μl of real time master mix (Ampilicon, Denmark). Thermal cycling was preceded at 95 ^°^C for 15 min followed by 40 cycles of PCR (95 ^°^C, 20 sec); annealing temperature was 61 ^°^C. Using the Pfaffle method (23), fold change in the expression of *CXCR4* was calculated relative to expression of the *HPRT* gene as a housekeeping gene. The sequence of *CXCR4 *is as follows: Forward: 5′ - CGC CAC CAA CAG TCA GAG 3΄ and Reverse: 5΄ AAC ACA ACC ACC CAC AAG TC - 3΄ .

***Statistical analysis***

 All data were representative of three independent experiments and calculated as the mean values ± standard deviation. Significant differences were evaluated by either the ANOVA test or the student t- test. Comparison of the data between normoxia and hypoxia was performed using independent t-test. One-way ANOVA was used to calculate the significance in each group. Statistics were calculated using Graph Pad prism6 software.* P-*value less than 0.05 was considered statistically significant.

## Results

***Total nucleated cell expansion***

**Figure 2:**
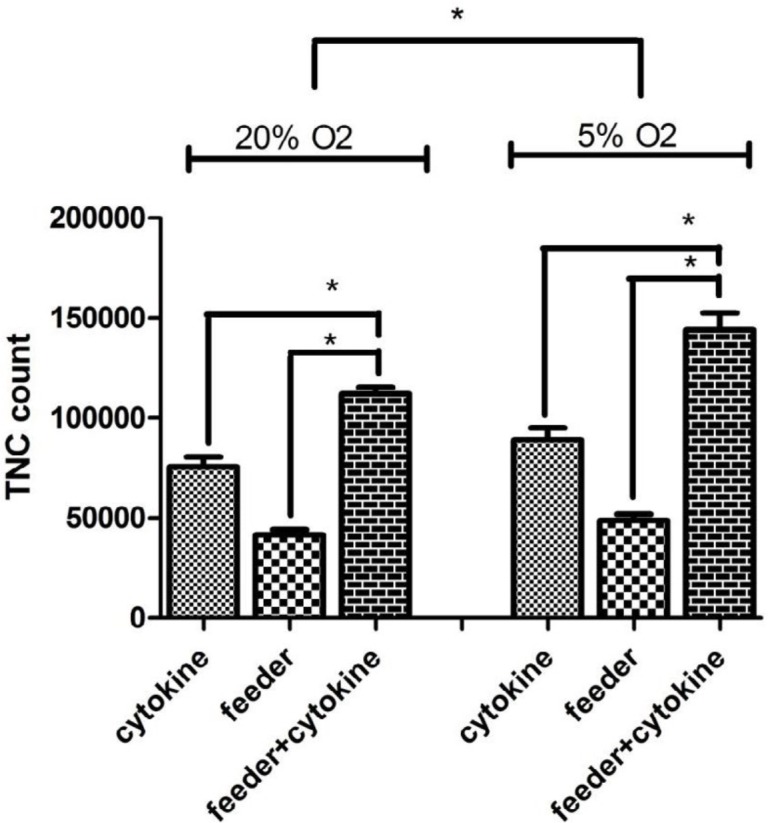
Total nucleated cells count in different culture conditions. CD34+ cells cultured in 6 different groups. After 7 days, total number of nucleated cells was counted. Data represent mean±SD from 3 independent experiments. Error bars represent SD.*P < 0.05: significant

To assess whether various culture conditions could accelerate cells expansion and preserve HSCs phenotype throughout the culture period, we analyzed the count increase of the cells after expansion. The trypan blue exclusion staining demonstrated >85 % viable cells both at 20% and 5 % O_2_ in different culture conditions. The initial cell count was 1×10^4^ and after 7 days culture in normoxia, the mean TNC count was 75600 ± 4900 in cytokine group, 41540±2660 in feeder group and 112000±3361 in feeder+cytokine group. In hypoxic cultures, the mean TNC count was 89000±6151 in cytokine group, 48700±3161 in feeder group and 144080±8518 in feeder+cytokine group. In all co-cultures on feeder layer, TNC increased significantly compared to without feeder culture conditions (N=3, *P*<0.05). In hypoxic conditions compared to normoxia, TNC increased significantly (*P*<0.05) (Figure 2).

***CD34+ cells expansion***

**Figure 3:**
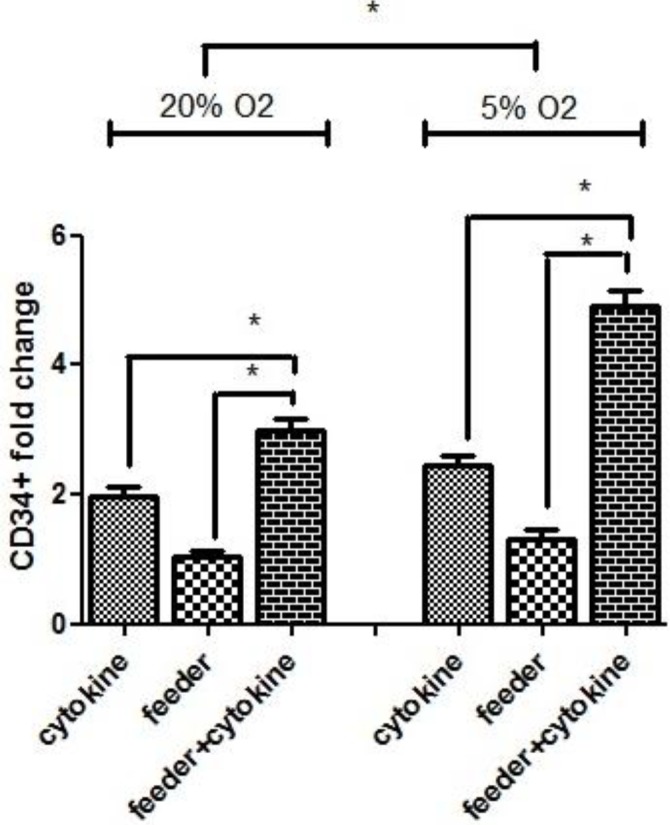
CD34+ cells fold change in different culture conditions; expanded CD34+ cells from 6 different culture conditions evaluated by flowcytometry. Data represent mean±SD from 3 independent experiments. Error bars represent SD. *P<0.05: significant

 CD34 as a HSCs marker was enumerated to quantify stem/progenitor cell population. CD34+ cell purity was evaluated by flowcytometry analysis using a FITC human CD34 antibody and was > 90 % in all cases on day 1 of purification. Figure 3 shows the fold change of CD34+ cells in different culture conditions after 7 days. The mean fold change of CD34+ cells in 20 % O_2_ tension was 1.96±0.15 in cytokine group, 1.04±0.08 in feeder group and 2.98±0.2 in feeder+cytokine group. In 5 % O_2_ tension, the mean fold change of CD34+ cells was 2.45±0.15 in cytokine group, 1.32±0.15 in feeder group and 4.91±0.23 in feeder+cytokine group. The mean fold change of CD34+ cells at day 7 in feeder+cytokine group was higher than cytokine group and feeder group (N=3, *P*<0.05). Our result showed that both culture on feeder layer and hypoxic environment had a significant effect on the outcome of the final CD34+ cells, and the collective impact of these two factors was remarkable (*P*<0.05).

In the feeder group without cytokine, TNC and CD34 + cell numbers increased up to 5 and 1.5 fold, respectively and cell viability remained 90% after 7 days. In both hypoxia and normoxia cultures, TNC count and CD34+ fold change were higher in cytokine groups compared to feeder group. (Figure 2, and 3).

**Figure 4:**
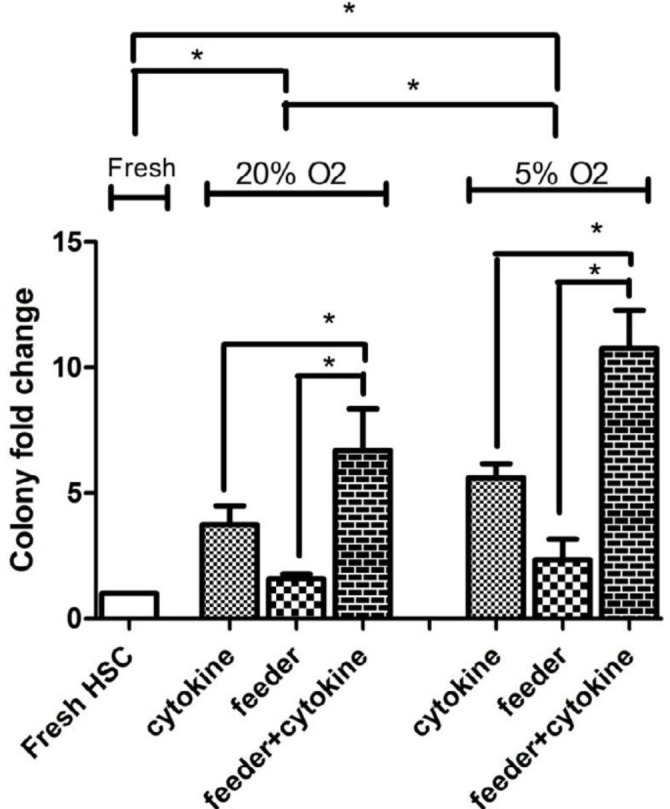
Colony fold change in different culture conditions. Results of clonogenic assay of cord blood CD34+ cells in different culture conditions plated after 14 days. The experiments performed in duplicate in 3 independent experiments. Error bars represent SD.*P < 0.05: significant

**Figure 5:**
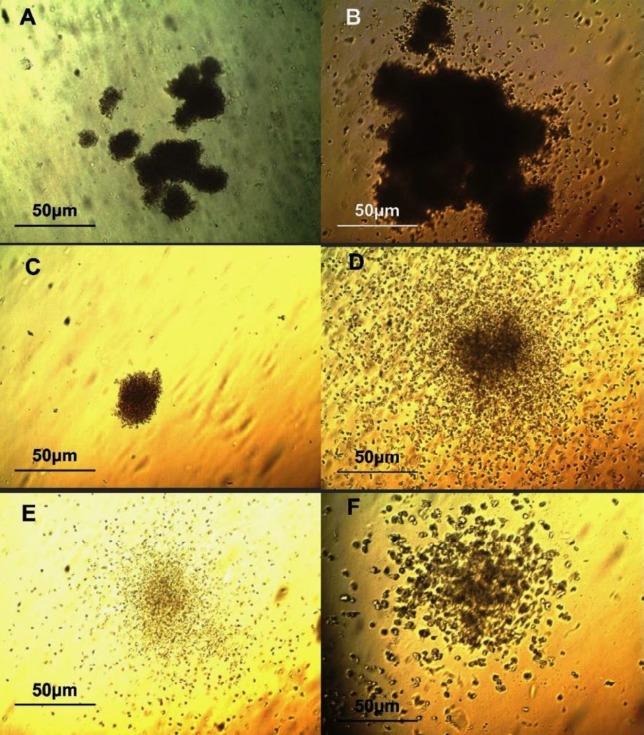
Morphology of colonies cultured 14 days in MethoCult H4434 classic with cytokine. (A) Burst forming unit-erythroid (BFU-E), (B) colony forming unit -granulocyte, erythroid, macrophage, megakaryocyte (CFU-GEMM), (C) colony forming unit -erythroid (CFU- E), (D) colony forming unit –granulocyte, monocyte (CFU-GM), (E) colony forming unit- granulocyte (CFU-G), (F) colony forming unit -monocyte (CFU-M), (scale bars: A–F, 50 μm)

**Figure 6:**
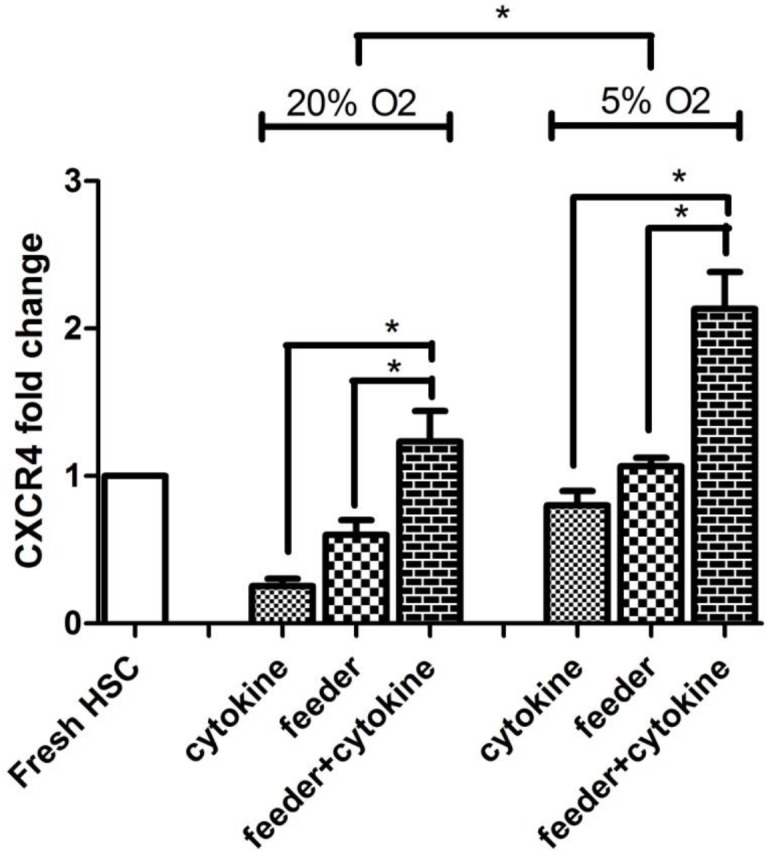
The mean fold change of CXCR4 mRNA expression in different culture conditions by real time PCR (N=3; *P<0.05: significant). Data are normalized to HPRT expression levels

**Figure 7:**
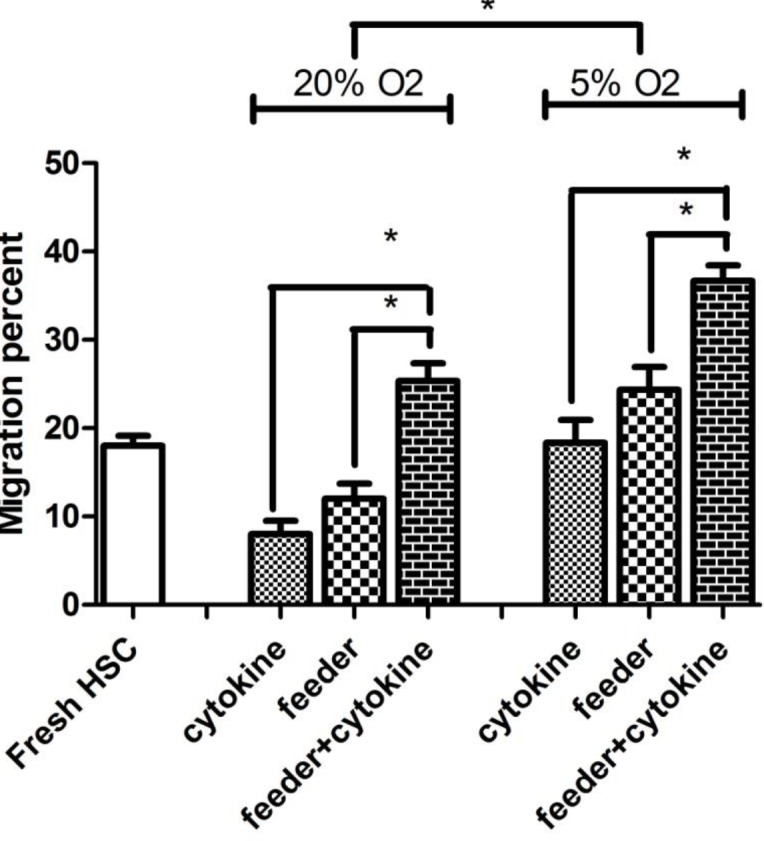
The mean percentage of migration toward stromal cell-derived factor 1 (SDF-1) in different culture conditions. The chemotactic effect of SDF-1 on CD34+ cells migration in the 6 different culture conditions after 4 hr. Results show mean percentage of migration from 3 independent experiments. Error bars represent SD. *P<0.05: significant

***Colony-forming assay***

The ability of forming colony was investigated on fresh CD34+ cells by methocult clonogenic assay (14 days) in 6 different culture conditions that were expanded for 7 days. The results showed that harvested cells from all culture conditions contained the ability to produce clonogenic progenitor cells: CFU granulocyte/ erythrocyte/ monocyte/ megakaryocyte (GEMM), CFU- granulocyte /monocyte (GM), CFU- granulocyte (G), CFU- monocyte (M), burst forming unit erythroid (BFU-E) and CFU- erythroid (E) similar to the fresh CD34+ cells. The expansion folds were calculated by comparing colonies obtained after 7 days with the colonies at the day 0 (Fresh HSC).

The mean CFU fold change of different culture conditions compared to fresh CD34+ cells was 3.7±0.7 in cytokine group, 1.6±0.2 in feeder group and 7±1.6 in feeder+cytokine group. In 5 % O_2_ tension, the mean CFU fold change was 5.6±0.5 in cytokine group, 2.3±0.8 in feeder group and 10.8±1.5 in feeder+cytokine group. Cultures in the presence of feeder layer and cytokine were able to maintain a higher clonogenic potential (*P*<0.05). There was higher colony fold change in hypoxia compared to normoxia. Data are shown in Figure 4 and colony morphology after 14 days of culture are shown in Figure 5.

***CXCR4 Gene expression***

In this study, expression of *CXCR4* in fresh CD34+ cells and harvested cells after 7 days was evaluated by real time PCR. The mean fold change ratio of *CXCR4* mRNA expression in normoxia was 0.25±0.05 in cytokine group, 0.6±0.1 in feeder group and 1.2±0.2 in feeder+cytokine group. In hypoxic culture, the mean fold change ratio was 0.8±0.1 in cytokine group, 1.06±0.06 in feeder group and 2.13±0.25 in feeder+cytokine group. 

We showed that in cytokine groups, *CXCR4* expression decreased rapidly in either normoxia or hypoxia, but in feeder groups without addition of cytokines, *CXCR4* better maintained. The highest *CXCR4* level was observed in feeder+cytokine groups. The results showed that *CXCR4* gene expression was sensitive to oxygen level and presence of MSC feeder layer (Figure 6) (N=3, *P*<0.05).

***HSC migration***

The mean percentage of migration after 4 hr was 18±2% in fresh CD34+ cells, but the mean percentage after 7 days was 8±2.6% in cytokine group, 12±3% in feeder group and 25.3±3.5% in feeder+cytokine group. In hypoxic culture, mean percentage of migration was 18.3±4.5% in cytokine group, 24.3±4.5% in feeder group and 36.6±3.05% in feeder+cytokine group. Control cultures without *SDF1* showed migration percentage less than 2%. The highest percentage of HSC migration was observed at feeder+cytokine group in 5% O_2_ (N=3, *P*<0.05) (Figure 7). The migration assay results agreed with the *CXCR4* gene expression results.

## Discussion

The major challenge in HSC expansion is still the finding optimal culture conditions concerning hematopoietic growth factor cocktails, cultures on feeder layer, and other stem cell niche requirements. Our experiments were carried out in stem line II serum-free culture media because in clinical use, serum-free media does not have concerns of serum, such as the transmission of infection by viruses/ prions, differences between batches (24), presence of stimulator or inhibitors of hematopoiesis, and finally, serum free-media contain chemically defined conditions (25).

Our finding showed that cytokine group without MSC feeder layer could efficiently increase expansion of CB-CD34+ stem cells. Several preclinical studies showed that SCF enhances homing capacity of CB cells (26). FLt3L induced short-term expansion and regulated adhesion molecules expression that contributed in proliferation and differentiation either directly or through the modulation of growth factor-induced signals (27). TPO is suggested to replace IL-3, IL-6 and G-CSF (28) and may inhibit apoptosis (29) and maintain the self-renewal of primitive HSCs by preventing telomere shortening (30).

Various cytokines have been shown to expand HSCs *ex vivo*, but were not sufficient to maintain and expand long-term repopulating (LT) HSCs, and prolonged culture resulted in an increase of more committed progenitor cells, which may dilute the number of true HSCs and reduce the efficiency of engraftment. Interaction between stem cells and their niches can modulate HSC functions (31, 32).

A more natural stem cell proliferation was achieved by culturing the stem cells on a layer of feeder cells of MSC (33,34). We showed that culturing in serum-free media with human BM-MSC-derived feeder layers supplemented with cytokine cocktail of SCF, TPO and FLt3L leads to higher expansion of CD34+ HSC from CB.

The mean fold changes of TNC, CD34+ cells and colony fold change at the 7^th^ day of expansion in the two cytokine groups with/without MSCs were higher than feeder group without cytokine. 

MSCs without cytokines maintain HSCs expansion at a low but a constant level during 7 days. In feeder group, TNC and CD34+ cell count were increased up to 5 and 1.5 fold, respectively, but cell viability was more than 90 %. It seems that cytokines and the growth signals secreted from BM-MSC might be transferred to the HSC through adhesion molecules. Song *et al*.(35) claimed that co-cultures of hematopoietic cells with BM-MSC without addition of cytokines were able to maintain repopulating HSCs for several weeks after transplantation .

The mean fold changes of TNC and CD34+ cells in feeder+cytokine group were higher than that in the cytokine group or feeder group alone. Consistent with our result, co-culture with MSC maintains HSC with a primitive immunophenotype (CD34+CD38- or CD133+CD38-) (36) and *ex vivo* contact with stromal layer preserves HSC (37, 38). Amirizadeh *et al.* (39) also showed higher expansion of HSCs in the cytokine culture with MSCs feeder layer compared to cytokine culture without MSCs, as well as the feeder culture without cytokine.

Several publications support the pivotal role of low O_2_ tension in the maintenance of stem cell homeostasis (similar to those in bone marrow) (13-17). It is accepted that residing in the hypoxic environment protects the stem cells from oxidative stress, and thus, the HSCs present in this environment have low reactive oxygen species (ROS) levels (40). We chose mild hypoxia (5% oxygen tension) because the relationship between the cycling/quiescence balance for HSCs was reported at 5 % O_2_, both *in vitro* and *in vivo* (41).

Higher TNC count and CD34+ fold change in mild hypoxia demonstrated that mild hypoxia stimulates expansion of the HSC. Several studies showed that culturing human CD34+ 38- HSCs in hypoxic conditions prior to transplantations improves their engraftment to nude mice (42, 43).

Our results showed that expanded cells in all 6 various culture conditions had the ability to produce clonogenic progenitor cells similar to the fresh CD34+ cells. To our knowledge, colony-forming potential depended on the number of HSCs and our results showed that CFC number in HSCs cultured on the MSCs feeder layer in the presence of cytokines at 5% O_2_ was higher than other culture conditions.

We assessed migration capacity of CD34+HSC, because a faster neutrophil recovery was observed in patients who received CD34+ cells with higher migration percentage *in vitro *(44). We showed a higher percentage of migration toward *SDF-1* in cells co-cultured with MSC at hypoxic culture conditions.

One of the most important interactions between MSCs and HSCs is the* CXCL12 *(*SDF-1*)/*CXCR4* axis. Studies reported that *in vivo* nestin+ MSC depletion rapidly reduces homing of hematopoietic stem/progenitor cells (10). Upregulation of *CXCR4* expression (45) or *CXCR4* overexpression by lentiviral transduction (46, 47) was shown to increase bone marrow homing of human CD34+ and CD34+CD38- cells in *NOD/SCID* mice. In clinical approach, decreased engrafting capability of CD34+ cells was reported with an absolute decrease in *CXCR4* expression (19); on the other hand, when the cells expressed higher amounts of *CXCR4*, lower doses of mobilized CD34+ cells/kg of body weight were needed (44). For these reasons, we analyzed the expression of *CXCR4* on hematopoietic CD34+ cells by real time PCR. Our results showed a significant increased expression of *CXCR4* on CD34+ cells in feeder+cytokine group in mild hypoxia that could play an important role in increased homing of HSC to the bone marrow.

Our results showed that despite higher expansion of HSC in cytokine group, *CXCR4* gene expression was lower in these group compared to Feeder group and Feeder+cytokine group. In accordance with our study, higher percentage of *CXCR4* negative cells was observed in only cytokine-supplemented cultures. The reason for downregulation of* CXCR4* expression could be fewer internal *CXCR4*, or an internalization of *CXCR4* (19).

We demonstrated statistically significant increased expression of* CXCR4* on CD34+ cells in mild hypoxia compared to normoxia (1.7-3.2 fold). Several studies demonstrated that *CXCR4* is the target gene of *HIF-1α *(48). Briefly, *HIF* proteins are members of the basic helix-loop-helix family. In normoxic atmosphere, *HIF-1α* is rapidly degraded by ubiquitin-proteasome pathway. The *prolyl hydroxylase domain protein 2 *(*PHD2*) enzyme can hydroxylate proline residues within *HIF-1α*. The activity of *PHD2* under hypoxic atmosphere is reduced and *HIF-1α* stabilizes and binds to hypoxia-regulated elements (HREs) in the promoters or enhancers of target genes (49). Therefore, targeting by the *HIF-1α* seems to be the reason for increased expression of *CXCR4* in hypoxia*.*

## Conclusion

In this study, we showed that harvested CD34^+^ cells were significantly increased when HSC were co-cultured with MSC and combinations of FLt3L, TPO and SCF cytokines, compared to cytokines or feeder layer culture alone. We demonstrated that, a decrease in O_2_ concentration is associated with increase of total nucleated cells and CD34+ cells, both in cultures and co-cultures. Beneficial effect of low O_2_ concentration could result from many factors regulated by O_2_ tension, such as cytokines/growth factor and their receptors, as well as other molecules in the *HIF-1*-binding site.

The results of this experiment support the conclusion that co-culture of CB-CD34+ and BM-MSC under mild hypoxia (5% O_2_) not only improves expansion of TNC and CD34+ cells but also increases the level of *CXCR4* gene expression and migration capacity of CD34+ cells. We concluded that several environmental factors, such as concentrations and combinations of cytokines, stromal interactions, and other stem cell niche conditions (such as O_2_ tension) are required for modulation of the quiescence/proliferation balances and homing capacity of HSC. We hope that these findings contribute to development of novel culture systems for the *ex vivo* expansion of CB-HSCs.

## References

[1] Wang LD, Wagers AJ (2011). Dynamic niches in the origination and differentiation of haematopoietic stem cells. Nat Rev Mol Cell Biol.

[2] Mendelson A, Frenette PS (2014). Hematopoietic stem cell niche maintenance during homeostasis and regeneration. Nat Med.

[3] Lilly AJ, Johnson WE, Bunce CM (2011). The haematopoietic stem cell niche: new insights into the mechanisms regulating haematopoietic stem cell behaviour. Stem Cells Int.

[4] Shizuru JA, Negrin RS, Weissman IL (2005). Hematopoietic stem and progenitor cells: clinical and preclinical regeneration of the hemato-lymphoid system. Annu Rev Med.

[5] Hordyjewska A, Popiołek Ł, Horecka A (2015). Characteristics of hematopoietic stem cells of umbilical cord blood. Cytotechnology.

[6] Jacobson CA, Turki AT, McDonough SM, Stevenson KE, Kim HT, Kao G (2012). Immune reconstitution after double umbilical cord blood stem cell transplantation: comparison with unrelated peripheral blood stem cell transplantation. Biol Blood Marrow Transplant.

[7] Majhail NS, Brunstein CG, Tomblyn M, Thomas AJ, Miller JS, Arora M (2008). Reduced-intensity allogeneic transplant in patients older than 55 years: unrelated umbilical cord blood is safe and effective for patients without a matched related donor. Biol Blood Marrow Transplant.

[8] Brunstein CG, Gutman JA, Weisdorf DJ, Woolfrey AE, Defor TE, Gooley TA (2010). Allogeneic hematopoietic cell transplantation for hematologic malignancy:relative risks and benefits of double umbilical cord blood. Blood.

[9] Tanavde VM, Malehorn MT, Lumkul R, Gao Z, Wingard J, Garrett ES (2002). Human stem-progenitor cells from neonatal cord blood have greater hematopoietic expansion capacity than those from mobilized adult blood. Exp Hematol.

[10] Mendez-Ferrer S (2010). Mesenchymal and haematopoietic stem cells form a unique bone marrow niche. Nature.

[11] Jing D, Fonseca AV, Alakel N, Fierro FA, Muller K, Bornhauser M (2010). Hematopoietic stem cells in co-culture with mesenchymal stromal cells - modeling the niche compartments in vitro. Haematologica.

[12] Majumdar MK, Thiede MA, Mosca JD, Moorman M, Gerson SL (1998). Phenotypic and functional comparison of cultures of marrow-derived mesenchymal stem cells (MSCs) and stromal cells. J Cell Physiol.

[13] Dellatore SM, Garcia AS, Miller WM (2008). Mimicking stem cell niches to increase stem cell expansion. Curr Opin Biotechnol.

[14] Hermitte F, Brunet GP, Belloc F, Praloran V, Ivanovic Z (2006). Very low O2 concentration (01%) favors G0 return of dividing CD34+ cells. Stem Cells.

[15] Tiwari A, Wong CS, Nekkanti LP, Deane JA, McDonald C, Jenkin G (2016). Impact of oxygen levels on human hematopoietic stem and progenitor cell expansion. Stem Cells Dev.

[16] Cipolleschi MG, Rovida E, Ivanovic Z, Praloran V, Olivotto M, Delo Sbarba P (2000). The expansion of murine bone marrow cells preincubated in hypoxia as an in vitro indicator of their marrow-repopulating ability. Leukemia.

[17] Ivanović Z, Bartolozzi B, Bernabei PA, Cipolleschi MG, Rovida E, Milenković P (2000). Incubation of murine bone marrow cells in hypoxia ensures the maintenance of marrow-repopulating activity together with the expansion of committed progenitors. Br J Haematol.

[18] Zhang CC, Sadek HA (2014). Hypoxia and metabolic properties of hematopoietic stem cells. Antioxid Redox Signal.

[19] Denning-Kendall P, Singha S, Bradley B, Hows J (2003). Cytokine expansion culture of cord blood CD34+ cells induces marked and sustained changes in adhesion receptor and CXCR4 expressions. Stem Cells.

[20] Asfour I, Afify H, Elkourashy S, Ayoub M, Kamal G, Gamal M (2017). CXCR4 (CD184) expression on stem cell harvest and CD34+ cells post-transplant. Hematol Oncol Stem Cell Ther.

[21] Ceradini DJ, Kulkarni AR, Callaghan MJ, Tepper OM, Bastidas N, Kleinman ME (2004). Progenitor cell trafficking is regulated by hypoxic gradients through HIF-1 induction of SDF-1. Nat Med.

[22] Yellowley C (2013). CXCL12/CXCR4 signaling and other recruitment and homing pathways in fracture repair. Bonekey rep.

[23] Pfaffl MW (2001). A new mathematical model for relative quantification in real-time RT–PCR. Nucleic Acids Res.

[24] Möbest D, Mertelsmann R, Henschler R (1998). Serum free ex vivo expansion of CD34(+) hematopoietic progenitor cells. Biotechnol Bioeng.

[25] Lebkowski JS, Schain LR, Okarma TB (1995). Serum-free culture of hematopoietic stem cells: a review. Stem Cells.

[26] Zheng Y, Sun A, Han ZC (2005). Stem cell factor improves SCID repopulating activity of human umbilical cord blood-derived hematopoietic stem/progenitor cells in xenotransplanted NOD/SCID mouse model. Bone Marrow Transplant.

[27] Oubari F, Amirizade N, Mohammadpour H, Nakhlestani M, Zarif MN (2015). The important role of FLT3-L in Ex vivo expansion of hematopoietic stem cells following co-culture with mesenchymal stem cells. Cell J.

[28] Levac K, Karanu F, Bhatia M (2005). Identification of growth factor conditions that reduce ex vivo cord blood progenitor expansion but do not alter human repopulating cell function in vivo. Haematologica.

[29] Goyama S, Mulloy JC (2013). Making Healthy Stem Cells: The New Role of TPO. Cell Stem Cell.

[30] Gammaitoni L, Weisel KC, Gunetti M, Wu KD, Bruno S, Pinelli S (2004). Elevated telomerase activity and minimal telomere loss in cord blood long-term cultures with extensive stem cell replication. Blood.

[31] Boitano AE, Wang J, Romeo R, Bouchez LC, Parker AE, Sutton SE (2010). Aryl hydrocarbon receptor antagonists promote the expansion of human hematopoietic stem cells. Science.

[32] Wilson A, Oser GM, Jaworski M, Blanco-Bose WE, Laurenti E, Adolphe C (2007). Dormant and self renewing hematopoietic stem cells and their niches. Ann N Y Acad Sci.

[33] Goncalves R, Lobato da Silva C, Cabral JM, Zanjani ED, Almeida-Porada G (2006). A Stro-1( + ) human universal stromal feeder layer to expand/maintain human bone marrow hematopoietic stem/progenitor cells in a serum-free culture system. Exp Hematol.

[34] Zhang Y, Chai C, Jiang XS, Teoh SH, Leong KW (2006). Co-culture of umbilical cord blood CD34 + cells with human mesenchymal stem cells. Tissue Eng.

[35] Song Y, Bahnson A, Hall N, Yu H, Shen H, Koebler D (2010). Stem cell traits in long term co-culture revealed by time-lapse imaging. Leukemia.

[36] Walenda TH, Bork S, Horn P, Wein F, Saffrich R, Diehlmann A (2010). Co-culture with mesenchymal stromal cells increases proliferation and maintenance of haematopoietic progenitor cells. J Cell Mol Med.

[37] Schmal O, Seifert J, Schäffer T (2016). E, Walter C. B, Aicher W.K, Klein G. Hematopoietic stem and progenitor cell expansion in contact with mesenchymal stromal cells in a hanging drop model uncovers disadvantages of 3D culture. Stem Cells Int.

[38] Chute JP, Saini AA, Chute DJ, Wells MR, Clark WB, Harlan DM (2002). Ex vivo culture with human brain endothelial cells increases the SCID-repopulating capacity of adult human bone marrow. Blood.

[39] Amirizadeh N, Oodi A, Nikougoftar M, Soltanpour MS (2013). Expression and promoter methylation changes of the P15INK4b during ex vivo cord blood CD34+ cell expansion following co-culture with mesenchymal stromal cells. Hematology.

[40] Jang YY, Sharkis SJ (2007). A low level of reactive oxygen species selects for primitive hematopoietic stem cells that may reside in the low-oxygenic niche. Blood.

[41] Guitart AV, Hammoud M, Dello Sbarba P, Ivanovic Z, Praloran V (2010). Slow-cycling/quiescence balance of hematopoietic stem cells is related to physiological gradient of oxygen. Exp Hematol.

[42] Danet GH, Pan Y, Luongo JL, Bonnet DA, Simon MC (2003). Expansion of human SCID repopulating cells under hypoxic conditions. J Clin Invest.

[43] Shima H, Takubo K, Iwasaki H, Yoshihara H, Gomei Y, Hosokawa K (2009). Reconstitution activity of hypoxic cultured human cord blood CD34-positive cells in NOG mice. Biochem Biophys Res Commun.

[44] Voermans C, Kooi ML, Rodenhuis S, van der Lelie H, van der Schoot CE, Gerritsen WR (2001). In vitro migratory capacity of CD34+ cells is related to hematopoietic recovery after autologous stem cell transplantation. Blood.

[45] Ratajczak MZ, Suszynska M (2016). Emerging strategies to enhance homing and engraftment of hematopoietic stem cells. Stem Cell Rev and Rep.

[46] Brenner S, Whiting-Theobald N, Kawai T, Linton GF, Rudikoff AG, Choi U (2004). CXCR4-transgene expression significantly improves marrow engraftment of cultured hematopoietic stem cells. Stem Cells.

[47] Kahn J, Byk T, Jansson-Sjostrand L, Petit I, Shivtiel S, Nagler A (2004). Overexpression of CXCR4 on human CD34+ progenitors increases their proliferation, migration, and NOD/SCID repopulation. Blood.

[48] Speth JM, Hoggatt J, Singh P, Pelus LM (2014). Pharmacologic increase in HIF1α enhances hematopoietic stem and progenitor homing and engraftment. Blood.

[49] Hirota K, Semenza GL (2005). Regulation of hypoxia-inducible factor 1 by prolyl and asparaginyl hydroxylases. Biochem Biophys Res Commun.

